# Flares in systemic lupus erythematosus: diagnosis, risk factors and preventive strategies

**DOI:** 10.31138/mjr.28.1.4

**Published:** 2017-03-28

**Authors:** Christina Adamichou, George Bertsias

**Affiliations:** 1 Department of Rheumatology, Clinical Immunology and Allergy, University Hospital of Heraklion, Heraklion, Crete, Greece,; 2 University of Crete Medical School, Heraklion, Crete, Greece

**Keywords:** systemic lupus erythematosus, disease activity, organ damage, outcome

## Abstract

Despite advances in the treatment, patients with systemic lupus erythematosus (SLE) often experience disease exacerbations (flares) of varying severity. Their diagnosis is primarily made on clinical grounds after exclusion of other diseases or disturbances, primarily infections, and can be assisted by the use of validated clinical indices. Serological tests such as serum complement fractions and anti-dsDNA autoantibodies, are helpful in monitoring SLE activity, but they lack high diagnostic accuracy. Flares are more frequent in patients with persistent immunological and clinical activity, and have been described as significant risk factor for development of irreversible end-organ damage. Accordingly, prevention of flares has been recognized as a distinct therapeutic target in SLE and involves adequate control of disease activity, use of hydroxychloroquine, maintaining immunosuppressive or biologic therapy for several years, and avoiding non-compliance issues. The future holds promise for the discovery of biomarkers that will accurately predict or diagnose SLE flares, thus allowing for the implementation of patient-tailored preventive strategies.

## INTRODUCTION

Systemic lupus erythematosus (SLE) is a chronic autoimmune disorder characterized by a broad spectrum of symptoms and manifestations, encompassing almost all organs and tissues. Typically, its natural history follows a relapsing-remitting course with highly variable outcome and significant morbidity. Although patient survival rates have improved considerably over the last decades, most likely due to early disease identification and treatment, recognition of milder forms of the disease, advances in medical therapy and better management of complications;^1^ still, the majority of SLE patients experience repeat exacerbations (flares) during the disease course,^2^ which may adversely impact on short- and long-term outcome. In this review, we discuss the diagnosis and classification of lupus flares and the challenges in differentiating them from common mimicking conditions. We summarize the evidence regarding the frequency, risk factors and prognostic implications of flares. Accordingly, prevention of flares represents a major target in the management of SLE and we conclude by describing available strategies for achieving this goal.

## SEARCH STRATEGY

We searched PubMed for English-language articles published between January 1, 2000 and June 1, 2016, using the following index terms: “lupus”, “SLE”, “flare”, “relapse”, “exacerbation” and “disease activity”. Additional articles were retrieved from the references included in those articles, and also from our personal knowledge of the subject. The tiles/abstracts and/or full-text were reviewed, and when relevant findings were reported, the article was considered.^3^

## DIAGNOSIS AND CLASSIFICATION OF SLE FLARES

In 2011, a working party organized by the Lupus Foundation of America defined a flare in a lupus patient as: ‘*a measurable increase in disease activity in one or more organ systems involving new or worse clinical signs and symptoms and/or laboratory measurements. It must be considered clinically significant by the assessor and usually there would be at least consideration of a change or an increase in treatment*.’’^4^ However, diagnosing a flare in SLE can be perplexing due to the multi-systemic nature of the disease and the low or modest specificity of its manifestations, which should be discriminated from other conditions such as infection, drug adverse events, and fibromyalgia. Consequently, prompt recognition and accurate definition of flares is important not only for evaluating drug responses and clinical outcomes in clinical and epidemiological studies, but also in everyday practice for guiding therapeutic decisions.

Various SLE flare definitions have been developed in the context of clinical trials and are generally based on one or more of the following parameters: a) increase in disease activity score assessed by a validated index, b) appearance of new or worsening of disease manifestations, (e.g., increase in proteinuria in the case of renal flares), c) change in the physician’s global assessment (PGA) scale towards more active/severe disease, and d) need for treatment intensification (e.g., an increase of steroid dosage) (**Tables 1** and **2**).^2,5,6^ Specifically, the Safety of Estrogen in Lupus Erythematosus National Assessment (SELENA)-SLE Disease Activity Index (SLEDAI) Flare Index (SFI) is a composite tool that incorporates changes in the SLEDAI, individual organ manifestations not captured by the SLEDAI, changes in the treatment and the PGA, as well as the need for hospitalization due to lupus exacerbation.^6^ The SFI is easy to use in routine clinical practice, classifies mild/moderate and severe flares, and has been extensively validated in numerous observational and therapeutic studies. Limitations include the lack of inclusion of immunosuppressive (e.g., mycophenolate) and biologic drugs (e.g., belimumab) that have been more recently introduced in the treatment of SLE, and that it is not sensitive in capturing organ-specific flares. Moreover, the original SFI does not discriminate between mild (often not clinically significant) and moderate flares, although this has been rectified in a revised version of the index (R-SFI).^7,8^ Another shortcoming of the SFI is that it may misdiagnose a flare on the basis of starting or switching immunosuppressive treatment to take advantage of its potential steroid-sparing effects or due to toxicity or inadequate clinical response.^9^

**Table 1.** Definitions of SLE flares according to existing validated indices^2,5,10^

Index	Definition(s)
PGA^1^	*Mild/moderate*: increase by ≥1.0 compared with the previous visit *Severe*: increase by ≥1.0 to ≥2.5
SLEDAI	*Mild/moderate*: increase by >3 *Severe*: increase by >10
SFI	*Mild/moderate*: 1) increase of SLEDAI by ≥3 points; and/or 2) new/worse skin, stomatitis, serositis, arthritis, fever; and/or 3) increase in PGA by ≥1.0; and/or 4) treatment intensification: increase in prednisone <0.5 mg/kg or added NSAIDs or hydroxychloroquine *Severe*: 1) increase of SLEDAI by >12; and/or 2) new/worse CNS involvement, vasculitis, glomerulonephritis, myositis, platelet counts <60,000/mm^3^, hemolytic anemia (hemoglobin <70 g/L), requiring doubling of prednisone dose or dose >0.5 mg/kg; and/or 3) need for hospitalization due to SLE; and/or 4) any manifestation requiring prednisone >0.5 mg/kg or new immunosuppressive therapy; and/or 4) increase in PGA to >2.5
BILAG	*Moderate*: increase from C, D or E to B score in any system *Severe*: increase to A score in any system
SLAM	Increase by ≥3
LAI	Increase by >0.26

1 PGA, Physician Global Assessment; SLEDAI, SLE Disease Activity Index; SFI, SELENA-SLEDAI Flare Index; NSAID, non-steroidal anti-inflammatory drugs; CNS, central nervous system; BILAG=British Isles Lupus Assessment Group; SLAM, SLE Activity Measure; LAI, Lupus Activity Index

**Table 2.** Definition of renal flares in SLE

**Parameter**	**Proteinuric flare**	**Nephritic flare**
Serum creatinine	Stable (<30% increase over baseline level)	*Mild/moderate*: stable (<30% increase over baseline level) *Severe*: ≥30% increase
Proteinuria	Increase to >2 g/24hr	*Mild*: increase to ≤2 g/24hr *Moderate/severe*: increase to >2 g/24hr
Hematuria	<10 rbc/hpf	*Mild*: ≥10 RBCs/hpf ^1^ if baseline levels were <10; or, increase by at ≥2 fold if baseline levels were ≥10 *Moderate/Severe*: ≥10 RBCs/hpf; or, increase if previously on partial response
Cellular casts	No change	Reappearance if previously on remission; or, an increase in number of cellular casts if previously on partial response

1 Red blood cells per high-power field

* Table modified from Illei G G, Takada K, Parkin D, Austin H A, Crane M, Yarboro C H, et al.^66^

Another validated instrument for diagnosing flares in SLE is the British Isles Lupus Assessment Group (BILAG) index and its revised version, BILAG 2004. The BILAG is a comprehensive, sensitive-to-change scoring system in which lupus activity in different organs/systems is documented separately and is based on *physician’s intent to treat*.^5^ Advantages include the analytic description of activity from different organs and that it incorporates varying levels of disease severity (for example, the percentage of body surface area affected by a lupus rash). Accordingly, activity from each organ is scored between “A” (severe) and “E” (absent). A moderate flare is defined as a new BILAG “B” score in at least two systems and severe flare as a new “A” score in any system.^7^ The main drawback of BILAG is that it is cumbersome to use in daily practice, since it requires training and familiarization with the glossary of definitions. Together, both the SFI and the BILAG have been recognized to lack either in sensitivity (i.e., failing to recognize milder exacerbations of disease activity) or specificity (i.e., diagnosing flares that may not always be clinically relevant).^10^ To some extent, these limitations can be overcome by the inclusion of PGA as part of the patient evaluation. Notwithstanding, the use of validated instruments for diagnosing, documenting and classifying flares in daily clinical practice is critical as it aids the objective monitoring of the disease status, has important prognostic implications and can guide therapeutic decisions.

### Flare mimics

Lupus flares often present with non-specific (e.g., hair loss) or constitutional symptoms that need to be differentiated from other causes; particularly fibromyalgia, drug reaction, infection, metabolic (e.g., iron deficiency) or endocrine disorder, and, less frequently, malignancy. Although the presence of objective signs of lupus activity (e.g., frank synovitis), especially when from multiple organs, increases the likelihood for underlying disease exacerbation; still, a detailed history, complete physical examination, and targeted laboratory work-up is required to exclude other causes. In particular, fever is a frequent manifestation of lupus flare but necessitates exclusion of possible infection, especially in patients receiving moderate-to-high doses of glucocorticoids, immunosuppressive or biologic therapy. The diagnosis of infection is supported by the presence of shaking chills, leukocytosis and/or neutrophilia (especially in the absence of steroid therapy), increased numbers of band forms or metamyelocytes on peripheral blood smear, and concomitant immunosuppressive treatment.^11^ Conversely, active SLE is suspected in cases of leukopenia (not explained by cytotoxic therapy), normal or only slightly increased C-reactive protein (CRP), and coexisting serological activity (low complement factors C3/C4, elevated anti-DNA titres). If fever persists despite treatment with prednisone >40 mg/day, it is likely that that fever is due to infection rather than SLE.

Several biomarkers have been evaluated as candidate tools for differentiation of SLE flares versus infections. C-reactive protein levels tend to rise significantly during active infections; whereas in the setting of a lupus relapse, elevation is usually mild or absent.^12,13^ However, this increase during infections may be attenuated in immunocompromised patients, especially in those on treatment with corticosteroids.^14^ On the other hand, elevated CRP levels can also be found in SLE patients with active serositis or synovitis and no evidence of infection, thus making CRP a marker with low diagnostic specificity.^15–17^

Serum procalcitonin (PCT) is another biomarker that increases in bacterial infections but remains low during viral infections and non-infectious inflammatory conditions, making it a potentially useful tool for distinguishing between serious infections and disease exacerbations in SLE.^18^ Specifically, low serum PCT levels (<0.17 ng/ml) had >90% negative predictive value for ruling out bacterial infection in SLE patients.^19^ A review of the literature published until 2014 indicated that raised PCT levels ≥0.5 μg/L should strongly suggest bacterial infection in the context of SLE.^20^ However, there is limited data regarding the diagnostic accuracy of PCT in case of hemophagocytic syndrome, a severe condition which can mimic or complicate both lupus flare and infection.

Another study suggests the use of the delta neutrophil index (DNI), which reflects the fraction of peripheral blood immature granulocytes, to differentiate infection from SLE flare.^21^ In this study, a DNI value >2.8% had 54% sensitivity and 88% specificity for diagnosing infection in febrile SLE patients. Together, despite encouraging preliminary results, aforementioned biomarkers will require further standardization and validation before they are introduced in daily clinical practice.

#### Serological tests in the diagnosis of lupus flare

Serum autoantibodies and complement factors are widely used for measuring activity and diagnosing flares in SLE.^22^ Antibodies to double-stranded DNA (anti-dsDNA) are found in approximately 50% of SLE patients,^23^ and their serum levels correlate with lupus activity, especially nephritis.^24–27^ However, the magnitude of this association varies significantly across different studies with positive likelihood ratios ranging from 0.88 to >10.^28^ Stably positive anti-dsDNA, even if at a high titre, have poor utility in predicting SLE flares; whereas changes in anti-dsDNA titres may be more informative.^29^ Increases in anti-dsDNA frequently (40–60%) precede disease exacerbations by a period of few weeks or months, especially in the context of renal involvement,^30–33^ suggesting that they could be used for preventing SLE flares by pre-emptive treatment.^34–36^ However, such a strategy carries the risk of overtreating patients with glucocorticoids and contributing to significant associated harms. Instead, patients with surges in anti-dsDNA titres should be monitored closely for the early recognition of objective features of active lupus.^37^ An increase in anti-dsDNA serum levels some weeks before an exacerbation followed by a moderate decrease at the time of the flare has also been described, especially in patients with lupus nephritis.^38^ This is consistent with the hypothesis that anti-dsDNA form immunocomplexes which are subsequently deposited in target tissues.^39,40^ However, this observation is of limited clinical value since frequent serological testing is costly and not all lupus exacerbations are accompanied by changes in anti-dsDNA titres.^38^

Antibodies to complement 1q (anti-C1q) have also been described as indicators of disease activity, especially nephritis, in SLE.^41,42^ Several studies have suggested a possible role of anti-C1q in early detection of renal relapse.^42, 43^ Matrat et al.^44^ further established an additional benefit in assessing titres of anti-C1q in conjunction with anti-dsDNA for diagnosing renal flares. Thus, the specificity and positive predictive value of increased anti-C1q/anti-DNA were 97% and 69%, respectively; the corresponding figures were 77% and 53% in patients with increased anti-dsDNA alone, and 84% and 56% with increased anti-C1q alone.^44^ At present, the clinical use of anti-C1q is limited due to lack of standardization of the ELISA assay and its cut-off levels.

Various proteins of the complement pathway are linked to SLE pathogenesis and have been used to measure its activity. Serum C4 (but not C3) levels tend to decrease approximately two months prior to clinical appearance of a renal flare, reflecting the early activation of the classical complement pathway. By the time of the flare, serum C3 concentrations are usually reduced, suggesting that the tissue damage may involve predominantly the alternative pathway of complement activation.^45^ Accordingly, serum C3 levels have been reported to be abnormally low around the time of flare at a higher prevalence compared to serum C4 (64% versus 36%, reaching 95% versus 55% in the case of renal flares). Together, serum C3 levels may be more sensitive and specific than C4 to diagnose an SLE flare, which has led to the suggestion of measuring only C3 – rather than both C3 and C4 – in monitoring disease activity.^46,47^

Although the concordance of serological and clinical activity has been well demonstrated in several studies, there is a subset (6–15%) of SLE patients who manifest prolonged persistent hypocomplementenemia and/or elevated anti-dsDNA antibody levels in the absence of clinical manifestations (so-called serologically active clinically quiescent disease).^48^ The management of these patients has been a subject of debate, as it has raised the concern of subclinical damage progression. However, this has not been demonstrated^49^ and therefore, therapeutics should be based upon clinical activity as discussed below.

The nucleosome, the fundamental unit of chromatin, has been considered the principal autoantigen in SLE^50^ and is the primary target of anti-dsDNA and anti-histone autoantibodies. Nucleosome-specific antibodies (anti-NCS), which recognize only quaternary epitopes of the nucleosome particle, rather than its individual components (dsDNA and histones), have been correlated with SLE disease activity, lupus nephritis and flare.^51^ Ng et al. reported that anti-NCS antibodies may help to predict future flares (especially nephritis) in the subgroup of serologically active (anti-dsDNA >50 units/ml) clinically quiescent patients.^52^ Others, however, have questioned their clinical utility,^53^ while additional issues include the lack of consensus regarding the preparation of nucleosomes and the measurement of anti-NCS antibodies using analytical assays.

Antibodies to the ribosomal P proteins (anti-P) have been associated with specific organ activity in SLE, namely renal,^54^ liver^55,56^ and central nervous system (CNS) disease, particularly psychosis.^57,58^ However, the frequency in which they occur is rather low and ranges 6–20% in different ethnic groups.^59^ Furthermore, a systematic analysis reported low sensitivity (24–27%) and modest specificity (80%) of anti-P antibodies in diagnosing active CNS lupus,^60^ and thus, their use in the clinical assessment of SLE remains elusive.

## HOW COMMON ARE FLARES IN SLE AND WHO IS AT RISK?

It has long been recognized that the majority of SLE patients experience alternative periods of active and inactive disease, whereas monophasic illness is far less common.^61^ The frequency of flares varies widely across different studies depending on the patient population characteristics (ethnicity, baseline disease activity and severity, immunological profile), the observation period and the flare definition. Nonetheless, it is estimated that approximately 20–25% of SLE patients will flare within 1–2 years and 40–66% within 5–10 years after achievement of a low disease activity or remission status.^48,62–65^ Seventy to eighty percent of the flares are of mild or moderate severity, with the remaining 20–30% be classified as severe. The most frequently involved organs are the mucocutaneous, musculoskeletal (arthritis), hematological, renal (30–40%) and immunology. Similarly, long-term observational studies and extension of controlled trials in lupus nephritis have shown that as many as 40–50% of patients with a previous response to immunosuppressive treatment will experience one or more renal relapses during follow-up,^66–70^ the majority (60–70%) of them being proteinuric.

From a clinical viewpoint, identifying SLE patients who are at greater risk to develop flares, especially severe flares, is important in designing and implementing preventive strategies. To this end, a number of predictive parameters have been described (**Table 3**).^2,48,62–64,67,71–74^ Specifically, demographic characteristics associated with increased risk for flares include African-American ethnicity (OR 1.8 compared to Caucasian ethnicity), disease onset ≤25 years (hazard ratio [HR] 2.1), and male gender.^63,64,72^ Patients with history of major organ disease, particularly nephritis (HR 4.8) and cytopenia, are also at higher risk. Persistent disease activity is another strong predictor with 42% increased risk per 1-unit rise in average SLEDAI-2K. In terms of specific organ involvement, neuropsychiatric (HR 2.5–3.1), renal (HR 2.0–4.8), and vasculitis (HR 1.7–1.8) exhibit the strongest associations.^62,72^ Immunological activity (i.e., reduction in serum C3/C4 and/or increases in anti-dsDNA titres) also contributes to the risk (OR 2.2–2.8) for succeeding disease relapse. Notably, the flare risk due to heightened disease activity seems to persist over a period of several months, since use of glucocorticoids and initiation or intensification of immunosuppressive therapy within the previous year have both been linked to increased odds for future disease relapse. Therapy-wise, evidence from observational and controlled studies shows that discontinuation or no use of hydroxychloroquine may result in increased risk (OR 2.5) for SLE flares, including renal flares.^75,76^ Finally, although an array of putative disease biomarkers are currently being examined in SLE, only serum BLyS (B Lymphocyte Stimulator) has been tested in the context of randomized controlled trials, where baseline concentrations ≥2 ng/ml were shown to predict (HR 1.5–1.9) severe flares over a period of one year.^72^

**Table 3.** Risk factors for disease flares in patients with SLE

**Risk factors**
African-American race
Male gender
Age of SLE onset ≤25 years
Major organ disease (major cytopenias, neuropsychiatric lupus, nephritis, vasculitis)
Persistent clinical disease activity
Immunological activity (low serum C3/C4, high anti-dsDNA)
Poor compliance to treatment
No use or discontinuation of hydroxychloroquine
Quick tapering or withdrawal of maintenance immunosuppressive treatment
Serum BLyS levels ≥2 ng/ml

## THE BURDEN AND PROGNOSTIC IMPLICATIONS OF FLARES IN SLE

Flares in SLE patients incur significant clinical and financial burden. Approximately 30–40% of disease exacerbations involve at least two different organs/domains; the mucocutaneous, musculoskeletal, immunology, hematological and renal being the most frequent ones.^77,78^ Most severe flares are preceded by moderate flares in the same organ/domain.^64^ Importantly, up to 20% of severe flares are accompanied by new-onset disease or worsening of previously afflicted major organ such as serous membranes, kidneys and CNS.^73^

As a result of both inflammation and the adverse effects caused by the medications (e.g., high-dose glucocorticoids) used to control disease activity, SLE patients who experience flares are prone to develop irreversible dysfunction and damage of end-organs. Evidence from observational studies shows that for every exacerbation of SLE, the risk of subsequent organ damage, assessed by the validated index SLICC/ACR Damage Index (SDI), increases by almost 2-fold.^79^ In a study of 135 SLE patients, major flares (defined as BILAG “A” activity flares) were strongly associated (OR 18.9) with organ damage accrual or death over the 5 ensuing years.^80^ Likewise, occurrence of renal flares, especially nephritic flares, has been related to increased risk of doubling serum creati-nine and developing chronic kidney disease.^68,69^ Thus, in patients with proliferative or membranous lupus nephritis who were followed for a minimum of 3 years, spending more than 30% of time in renal flare was a powerful predictor (OR 20) for new-onset chronic kidney disease.^70^

Flares in SLE are critical from a cost-of-illness perspective and have been recognized as a major driver of increased direct healthcare cost. In the Systemic Lupus Erythematosus Cost of Care in Greece Study (LyCOS) of active SLE patients on treatment, severe flares (defined by modified SFI) contributed to 124% increase in the direct cost per annum.^81^ This excess was largely explained by the increased number of patient visits to health care providers, increased hospitalizations and use of medications by flaring compared to non-flaring patients. Importantly, flares have significant adverse impact on work productivity and other sources of indirect healthcare cost.^82^

## STRATEGIES FOR PREVENTING FLARES IN SLE

In view of the previous lines of evidence, prevention of flares, especially severe flares, is critical to ensure a better prognosis of SLE patients. Indeed, this is emphasized in the recently issued ‘treat-to-target’ recommendations for SLE.^83^ Accordingly, stabilization of the disease and minimization of the risk for flares should be viewed as a separate therapeutic goal, in adjunction to attaining low disease activity or remission.

Considering the well-recognized relationship between persistent disease activity and the risk for further worsening of SLE, the importance of tight disease control cannot be overemphasized. Of note, it is currently not recommended that treatment be escalated in cases of clinically quiescent but serologically active lupus, since this strategy carries the risk for over-treating patients with glucocorticoids.^83^ Instead, these patients should be monitored for the early identification of objective signs of disease exacerbation.

To date, there is limited evidence regarding the efficacy of different immunosuppressive agents in preventing SLE flare-ups. Azathioprine has been compared against ciclosporin in active SLE requiring ≥15 mg prednisolone/day with comparable results in reduction of disease activity and prevention of flares.^84^ In a randomized controlled study, patients with inactive disease who continued treatment with hydroxychloroquine had 74% lower risk for developing severe flares compared to their littermates who discontinued the drug.^76^ A similar protective effect of hydroxychloroquine has been demonstrated in patients with stable lupus nephritis on maintenance treatment.^75^ A number of different mechanisms of action have been suggested for antimalarials, including inhibition of the interaction between antigen-presenting cells and T-cells by disrupting the recycling of proteins in the cellular lysosomes and subsequently inhibiting the formation of major histocompatibility complex class II molecules from peptides, antagonizing endosomal Toll-like receptor-mediated immune responses and production of interferon-α and other inflammatory cytokines (reviewed in Wallace D J, et al.^85^). Apart from its immunomodulatory properties, hydroxychloroquine (and antimalarials in general) seem to exhibit also anti-thrombotic and anti-lipidemic effects. These effects, along with the long-term safety of the drug, have led to the suggestion of continuing it lifelong in patients with SLE, including during the period of pregnancy and lactation.^86^

More recently, belimumab has emerged as a biologic agent capable of stabilizing SLE activity. When added to the conventional treatment in patients with active moderate-to-severe lupus, belimumab leads to significantly reduced (by 36%) risk of major flare-ups over a period of one year.^87^

With regards to lupus nephritis, a controlled study in Caucasian patients with class III–IV lupus nephritis showed that maintenance treatment with azathioprine was as efficacious as mycophenolate mofetil in preventing renal flares and development of end-stage renal disease over a period of 10 years.^88^ Conversely, in the racially mixed Aspreva Lupus Management Study, maintenance treatment with mycophenolate mofetil was associated with significantly fewer renal relapses compared to azathioprine over a period of 3 years.^89^ Together, mycophenolate mofetil may be preferred as maintenance regimen in patients with more severe (clinically or histologically) lupus nephritis, black or Hispanic ethnicity, or when mycophenolate was used as induction regimen.

Optimum immunosuppressive drug tapering and withdrawal is also crucial for reducing the risk of SLE flare-ups. A large observational study in the Toronto Lupus Clinic showed that absence of serological activity and gradual dose tapering (by maximum 25%) of the immunosuppressant(s) were predictors of relapse-free drug withdrawal.^90^ Likewise, longer duration of immunosuppressive treatment and of renal response both correlate with higher odds for successful drug withdrawal in patients with lupus nephritis.^75^ In line with this, discontinuation or switching from mycophenolate to less potent agents such as azathioprine or calcineurin inhibitors, earlier than 18–24 months after attaining renal response, carries almost 2-fold increased risk for subsequent flare.^91,92^ Consequently, although the optimal duration of maintenance therapy in SLE has not been definitely established, we generally recommend a period of at least 2 to 3 years (5 to 7 years in lupus nephritis).

Finally, treating physicians should pay special consideration to any drug non-adherence issues and assess potential contributing factors. It is estimated that less than 25% of SLE patients have a drug adherence rate ≥80% of the time,^93^ and non-compliance to lupus treatment has been associated with increased risk of flares-ups and emergency care utilization.^94,95^

## CONCLUSIONS

Despite advances in the treatment, a significant proportion of SLE patients is prone to manifest one or more disease exacerbations which incur significant burden and may impact adversely on long-term outcome. The risk is particularly higher in context of continuous, uncontrolled serological and clinical disease activity, especially from major organs. Apart from targeting low disease activity or remission, physicians looking after SLE patients should therefore consider strategies for preventing disease exacerbations, particularly maintaining effective immunosuppressive and biological therapies at well-tolerated doses and for adequate time periods. For the future, we eagerly await the establishment of biomarkers capable to predict future flares with high accuracy, thus enabling the implementation of patient-tailored preventive strategies.

## ABBREVIATIONS:

ACR: American College of Rheumatology
BILAG: British Isles Lupus Assessment Group
BLyS: B Lymphocyte Stimulator
C1q: complement 1q
CNS: central nervous system
CRP: C-reactive protein
dsDNA: double-stranded DNA
HR: hazard ratio
NCS: nucleosome
OR: odds ratio
PCT: procalcitonin
PGA: physician’s global assessment
R-SFI: Revised SFI
SDI: SLICC/ACR Damage Index
SELENA: Safety of Estrogen in Lupus Erythematosus National Assessment
SFI: SELENA-SLEDAI Flare Index
SLE: systemic lupus erythematosus
SLEDAI: SLE Disease Activity Index
SLICC: Systemic Lupus International Collaborating Clinics
